# Reference values of nonword repetition test for Brazilian Portuguese-speaking children

**DOI:** 10.1590/S1678-77572009000700011

**Published:** 2009

**Authors:** Simone Rocha de Vasconcellos HAGE, Márcia Aparecida GRIVOL

**Affiliations:** 1 PhD, Speech Language Pathologist, Assistant Professor, Department of Speech-Language Pathology and Audiology, Bauru School of Dentistry, University of São Paulo, Bauru, SP, Brazil.; 2 Speech Language Pathologist, Graduate student, Department of Speech-Language Pathology and Audiology, Bauru School of Dentistry, University of São Paulo, Bauru, SP, Brazil.

**Keywords:** Memory, Language development, Speech-language pathology

## Abstract

Evaluation of the phonological working memory (PWM) through repetition of nonwords can provide important information on the linguistic abilities of children, thus differentiating those with and without communication disorders. Objective: The aim of this study was to obtain reference values in the Nonword Repetition Test (NWRT) in order to investigate the performance of children without language disorders concerning this type of memory. Material and Methods: The study was conducted on 480 normal children of both genders aged 4 years to 8 years and 11 months, attending preschool and elementary school. The NWRT consisted of repeating 20 (children up to 4 years) or 40 (for children aged 5 years or more) invented words with 2 to 5 syllables. The results were subjected to descriptive statistical analysis. Comparison between ages and between the number of syllables in nonwords was performed by the Tukey's multiple-comparison test and one-way analysis of variance, at a significance value of p<0.05. Results: There was statistically significant difference (p<0.05) in performance between children of different age groups, except between 7- and 8-year-olds. The analysis also showed statistically significant difference (p<0.05) in the number of syllables between the different age groups. Conclusions: The reference values obtained indicated an improvement in performance with the increase of age of children, indicating an improvement in the storage of verbal material in the PWM. The performance was worsened with the increase in the number of syllables in words, demonstrating that the greater the number of syllables, the greater is the difficulty in storing verbal material.

## INTRODUCTION

The Psycholinguistic Model (PLM) has decisively influenced the way to assess and treat language disorders in the last decade^8^. This model has been proven efficient because it explains how human beings process information coming to their senses, access the words stored in their lexicon and use the mental representations that encode information, thus understanding the nature of language disorders^6^^,^^9^^,^^19^^,^^23^. This model considers all processes involved in the act of communicating, from the primary level, involving the input and output of verbal information, up to the third, which corresponds to the level of cognitive operations of more complex language^17^.

These processes include the working memory, which plays a significant role in the maintenance of thinking and learning, verbal comprehension and lexicon access^11^^,^^14^^,^^16^. It is a system for processing and storing information on a short-term basis, organized into four components, namely the central executive, two work subsystems - the phonological and visuospatial loop -, and the episodic buffer^2^^,^^5^^,^^8^. The phonological loop stores and manipulates material based on speech and has two components: the phonological storage, which receives information through direct (auditory presentation) and indirect ways (visual presentation); and the reverberation process or subvocal test, which occurs serially in real time and acts to restrain the natural decay of phonological storage. One of the primary functions of the phonological loop or phonological working memory (PWM) is to store unfamiliar sound patterns, until a record of more permanent memory becomes consistent^1^^,^^4^.

The PWM has a fundamental role in acquiring language skills in children^8^^,^^13^ and its deficit has been suggested as the origin of linguistic difficulties in children with specific language impairment^1^^,^^15^^,^^16^^,^^18^^,^^24^^,^^25^.

In the clinical context, the PWM is evaluated by two procedures: digit span (repeating sequences of numbers in direct and inverse order) and repetition of words or nonwords (NW). The repetition of NW is indicated as a more reliable test for the PWM, because the verbal material input is unknown and hence not subject to lexical influences^3^^,^^4^^,^^10^^,^^21^.

Thus, considering the lack of instruments based on the Portuguese language for assessment of the PWM, the objective of this study was to obtain reference values for the Nonword Repetition Test (NWRT), investigating if there are differences in the performance of children without language disorders in different age groups, as well as if the increase of syllables of nonwords impairs their repetition.

## MATERIAL AND METHODS

The study was conducted on 537 children aged 4 years to 8 years and 11 months, of both genders, being 274 girls and 263 boys. Fifty-seven children were excluded due to the detection of problems in oral or written communication during sample selection. Thus, the study involved a final sample of 480 children, 231 boys and 249 girls, attending preschools and elementary schools in the São Paulo state countryside, according to the following inclusion criteria: no history of deficits in oral and written language, as reported on interviews with parents and teachers, who answered a questionnaire containing questions to check if the child had communication, hearing or school disturbances; phonological system compatible with chronological age, as assessed by the Task of Phonology of the Test of Children Language (ABFW)^26^; and, for children in the literacy process, punctuation appropriate to the age and schooling on the subtest of reading of the TDE - School Performance Test ^22^. Informed written consent approved by the local Institutional Review Board was obtained from patients regarding the specific procedure and the use of their data for research purposes.

For the NWRT^12^ (Appendix 1), all 480 boys and girls enrolled in the study were asked to repeat either 20 (children up to 4 years) or 40 (for children aged 5 years or more) invented words with 2 to 5 syllables. The NWRT was created based on the phonological structure of Portuguese language spoken in Brazil. It is divided in two parts, the first for children aged 3 and 4 years, consisting of 20 invented words with Portuguese phonemes, and the second for individuals above 5 years of age, consisting of 40 invented words with Portuguese phonemes, both containing sequences of 2 to 5 syllables. All invented words were paroxytone, because most words in Portuguese are also paroxytone, and were prepared containing different orders of the following phonemes: 6 occlusive (/ p /, / t /, / k, / b /, / d /, / g /), 3 nasal (/ m /, / n /, / η /), 6 fricative (/ f/, / v /,/]/,/ ς /, s /, / z /) and 2 liquids (/ I /, / R/), as well as 5 closed vowels (/ a /, / e /, / i /, / o /, / u /). The syllabic pattern used for children aged 3 and 4 years was C + V (C = consonant, V = vowel) and V + C; and for those above 5 years the pattern was C + V, V + C, C + V + C, C + C + V. The nonwords were prepared with the aid of combinatorial analysis, and the phonemes were combined in different positions in the nonwords, namely in the beginning, middle and end.

The list of nonwords was applied without visual clues, in the same vocal intensity, by a single examiner. The instructions were clearly provided to enhance the understanding: “I speak and you repeat” or “You speak after me”, “Now we are going to play ‘follow the leader’, the leader will speak words that do not exist and you will repeat them”. The child was scored 2 (two) points when the nonwords were repeated correctly in the first time, 1 (one) point when they were repeated correctly in the second time, and 0 (zero) point when the child was unable to repeat the nonwords correctly in two attempts.

The results were subjected to descriptive statistical analysis. Comparison between ages and between the number of syllables in nonwords was performed by the Tukey's multiple-comparisons test and one-way analysis of variance, at a significance value of p<0.05.

## RESULTS

The results showed statistically significant difference in performance between children of different age groups, except between seven and eight years (4 years <5 years <6 years < 7 years = 8 years).

The results showed that the performance was statistically different depending on the number of syllables of nonwords (F=206.1, p<0.001). The greater the number of syllables in nonwords, the worse was the children's performance in their repetition.

## DISCUSSION

The achievement of reference values for national evaluation tools is fundamental for the advancement of research in Brazil, particularly in the area of language, since the culture and language structure are important variables when testing cognitive and linguistic abilities^20^.

The instrument of this study was designed in accordance with the structure of the Brazilian language spoken in Brazil in order to obtain indices that can be used as reference for the evaluation of children with language problems, since lexical, syntactic and phonological difficulties have been related to deficits in PWM^1^^,^^16^^,^^25^. The PWM formed the theoretical basis for construction of this instrument because it allows the establishment of hypotheses on the mechanisms underlying the development of language - both in normal and pathological operations - and proposes strategies for the assessment and intervention that consider the various cognitive processes underlying the processing of linguistic information, such as PWM^6^^,^^9^^,^^19^^,^^23^.

The choice of tests involving the repetition of nonwords was based on studies^3^^,^^4^^,^^10^^,^^21^ that reported that the skills of PWM are more reliably assessed by repetition of this index, because the verbal material presented is not subject to lexical influences. The repetition of nonwords by children requires a connection between their system of perceptual analysis and phonological planning, and the perceptual analysis provides the sequence of phonemes that cannot be generated in the lexicon^10^.

The descriptive measures obtained in this study showed that, with the increase in age, children were more efficient in the accomplishment of NWRT, with progressive scores in the median and minimum value (Table 1). There was statistically significant difference between the performances of children of different age groups, except between 7- and 8-year-olds (Table 1), although the performance of eight-year-old children was on top of most descriptive measures. The expansion of memory with age is attributed to the increased speed of “subvocal recall” and is well related with the increase in language skills, typical of child development^8^^,^^13^. It is necessary to verify the age from which this performance is in decline, because seniors have memory decline, including in the verbal aspect^27^.

**Table 1:** Descriptive measures of the performance of children in the Nonword Repetition Test, considering the total scores obtained

Age	Subjects	Mean	Median	Minimum	Maximum	Lowest quartile	Superior quartile	Standard deviation
4 years old	106	34^A^	34	22	40	31	37	3.89
5 years old	94	58^B^	58	37	80	52	67	9.28
6 years old	80	68^C^	70	47	79	65	73	7.93
7 years old	117	74^D^	74	60	80	72	76	4.05
8 years old	83	74^D^	76	61	80	72	78	4.62

* Ages with the same letter in the mean are not statistically different.

**Figure 1:**
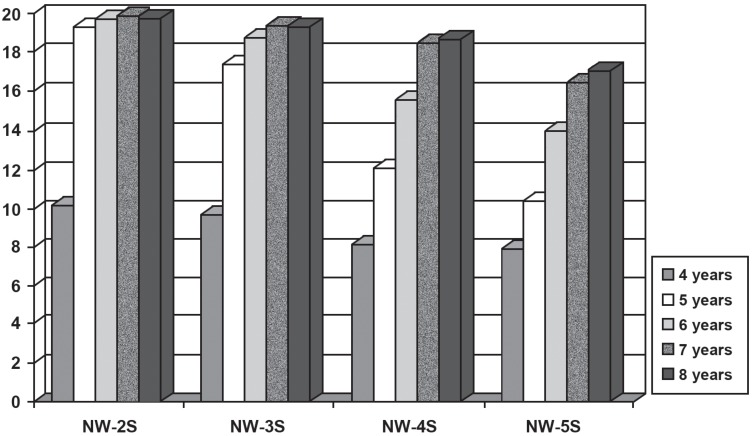
Descriptive measures of the performance of children aged 4 to 8 years according to the variable number of syllables. Legend: NW- nonword; S- syllable

**Figure 2:**
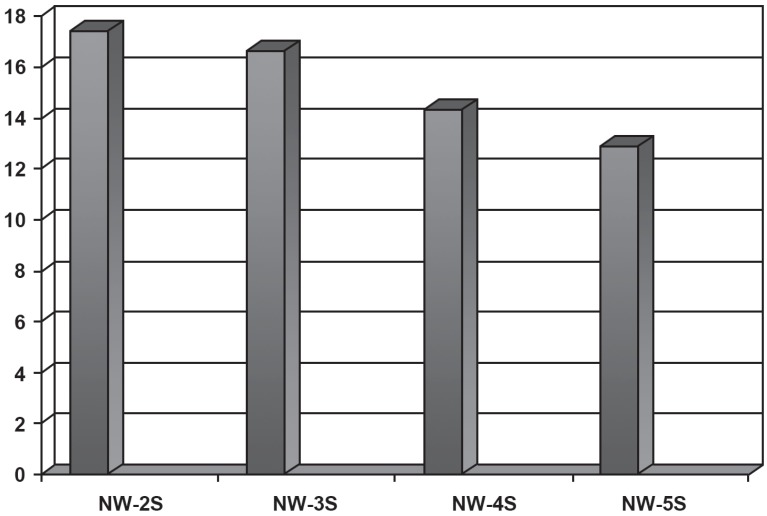
Comparative measurements between the numbers of syllables of nonwords. Legend: NW- nonword; S- syllable

Regarding the comparison between the number of syllables in nonwords, the results showed statistically significant difference between all of them (two syllables> three syllables> four syllables> five syllables) for the different age groups (Figure 1). The findings are consistent with the study of Santos and Bueno^21^ (2003), who found that the extent of nonwords is reflected in the subvocal test component of the MPWM, since the children's performance decreased as the number of syllables of nonwords increased. Thus, the greater the number of syllables, the greater the difficulty in storing verbal material in the memory (Figure 2).

## CONCLUSIONS

The reference values obtained indicated that the performance improves with the increase in age of children, indicating an increase in the storage of verbal material in the phonological working memory. There was worsening of the performance with the increase in the number of syllables in nonwords, demonstrating that the difficulty in storing the verbal material increased with the increase of the number of syllables. The results of this study may serve as parameters in the evaluation of children with language disorders and aid in diagnosing the nature of the possible linguistic deficit.
